# Setting up *Agrobacterium tumefaciens*-mediated transformation of the tropical legume *Aeschynomene evenia*, a powerful tool for studying gene function in Nod Factor-independent symbiosis

**DOI:** 10.1371/journal.pone.0297547

**Published:** 2024-04-16

**Authors:** Pierre Tisseyre, Fabienne Cartieaux, Nathalie Chabrillange, Djamel Gully, Valérie Hocher, Sergio Svistoonoff, Hassen Gherbi

**Affiliations:** 1 IRD (French National Research Institute for Sustainable Development), UMR QualiSud, IRD-MONTPELLIER, Montpellier, France; 2 IRD (French National Research Institute for Sustainable Development), UMR PHIM (Plant Health Institute of Montpellier), Montpellier, France; 3 Laboratoire commun de Microbiologie IRD/ISRA/UCAD, Centre de recherche de Bel Air, Dakar, Sénégal; University of Balochistan, PAKISTAN

## Abstract

Most legumes are able to develop a root nodule symbiosis in association with proteobacteria collectively called rhizobia. Among them, the tropical species *Aeschynomene evenia* has the remarkable property of being nodulated by photosynthetic *Rhizobia* without the intervention of Nod Factors (NodF). Thereby, *A*. *evenia* has emerged as a working model for investigating the NodF-independent symbiosis. Despite the availability of numerous resources and tools to study the molecular basis of this atypical symbiosis, the lack of a transformation system based on *Agrobacterium tumefaciens* significantly limits the range of functional approaches. In this report, we present the development of a stable genetic transformation procedure for *A*. *evenia*. We first assessed its regeneration capability and found that a combination of two growth regulators, NAA (= Naphthalene Acetic Acid) and BAP (= 6-BenzylAminoPurine) allows the induction of budding calli from epicotyls, hypocotyls and cotyledons with a high efficiency in media containing 0,5 μM NAA (up to 100% of calli with continuous stem proliferation). To optimize the generation of transgenic lines, we employed *A*. *tumefaciens* strain EHA105 harboring a binary vector carrying the hygromycin resistance gene and the mCherry fluorescent marker. Epicotyls and hypocotyls were used as the starting material for this process. We have found that one growth medium containing a combination of NAA (0,5 μM) and BAP (2,2 μM) was sufficient to induce callogenesis and *A*. *tumefaciens* strain EHA105 was sufficiently virulent to yield a high number of transformed calli. This simple and efficient method constitutes a valuable tool that will greatly facilitate the functional studies in NodF-independent symbiosis.

## Introduction

Nitrogen is a crucial element limiting plant production and nowadays more than 40% of the world’s population is dependent on synthetic nitrogen fertilisers derived from fossil fuels which allowed a dramatic increase of global agricultural productivity [1]. However, even today, a substantial portion of available nitrogen in agrosystems originates from a natural process called biological nitrogen fixation. This process is primarily carried out by a limited number of plant species capable of forming highly effective symbiotic associations with diazotrophic bacteria called Root Nodule Symbiosis (RNS). Within these plant species, most of members of the legume family are capable of engaging in RNS by interacting with proteobacteria collectively called *Rhizobia*.

Legume-rhizobia symbiosis has been extensively studied in the model species *Medicago truncatula* and *Lotus japonicus*, which helped deciphering several actors controlling the establishment and functioning of the symbiotic interaction [2]. In these 2 species, the infection process and the formation of nodule primordia are initiated by bacterial signaling molecules called Nod Factors (NodF = lipo-chitooligosaccharides). *Rhizobia* infection occurs intracellularly through root hair-constructed infection threads guiding bacterial entry to the developing nodule. Conversely, much less is known about molecular mechanisms leading to nodulation through intercellular infection-entry mode. This alternate way is considered simpler and is encountered in 25% of legume genera [3], often in tropical species. This is the case of Dalbergieae clade including *Aeschynomene* species, some of which are remarkable because they are nodulated by photosynthetic *Bradyrhizobium* without the intervention of NodF [4]. This original symbiotic mechanism commonly referred as the NodF-independent symbiosis, was studied in *Aeschynomene evenia* [5, 6]. Like other *Aeschynomene* spp, it is phylogenetically close to the cultivated peanut (*Arachis hypogaea*) but distant to *M*. *truncatula* and *L*. *japonicus*. *A*. *evenia* was established as a valuable working model [7] and genomic resources have been recently generated including a high-quality genome sequence [8] and a gene expression atlas. Furthermore, a comprehensive examination of symbiotic genes revealed either the absence or the lack of expression of crucial genes involved in NodF perception and intracellular infection while the downstream components of the NodF signaling pathway were expressed [1, 6] More recently, the screening of *A*. *evenia* nodulation mutants confirmed the involvement of downstream components of the NodF signaling pathway [8].

Progresses on gene discovery and characterization in RNS in the model species *M*. *truncatul*a and *L*. *japonicus* were made possible thanks to the development of genetic resources together with the genetic engineering approaches including efficient plant tissue regeneration and *A*. *tumefaciens*-mediated transformation. This implies the development of routine plant tissue culture and transformation protocols, the identification of a suitable selection marker and a reliable *in vitro* regeneration technique [9–12]. Thus, powerful genetic approaches based on loss-of-function (knock down using RNAi, knock out) or gain-of-function (complementation, ectopic expression, overexpression) strategies were used to demonstrate the biological function of several symbiotic genes.

In contrast, our understanding of NodF-independent symbiosis is very limited [8, 13], [13]. In *Aeschynomene*, investigating the biological functions of symbiotic genes relies on *Agrobacterium rhizogenes*-mediated transformation, which enables the generation of composite plants harboring transgenic hairy roots [14]. While this approach has facilitated RNAi-based functional studies [15, 16], it does possess some limitations. The transformed plants are chimeric, cannot be maintained, re-transformed or crossed and transgenic roots exhibit altered phenotypes [17]. One of the main challenges to conduct robust functional studies is the availability of efficient plant tissue culture methods and *A*. *tumefaciens*-based transformation protocols. Although there have been reports of *in vitro* regeneration of *Aeschynomene* plants [18], the regeneration of transgenic plants is particularly challenging due to species-specific properties that are often genotype-dependent [19].

In this study, we report the development of a simple and efficient *A*. *tumefaciens*-mediated transformation method allowing the generation of transgenic *A*. *evenia* plants within 4 months. By testing different compositions of tissue culture media, we defined the optimal conditions suitable for the genetic transformation, callus induction and plant regeneration. This new procedure enables a wide range of possibilities, facilitating molecular studies of NodF-independent symbiosis.

## Material and methods

### Biological material

#### Plant material

*Aeschynomene evenia* accession CIAT22838 (Malawi) was obtained from CIAT (Columbia). Cotyledons, epicotyl and hypocotyls were used as starting explants for regeneration assays, while only epicotyls and hypocotyls were used for the transformation procedure.

#### Bacterial strains

*Agrobacterium tumefaciens* strain EHA105 [20] harboring the binary vector pCambia5300 mCherry [21] was used for transformation experiments.

### Germination of *A*. *evenia* seeds

Seeds of *A*. *evenia* were scarified for 40 min with concentrated (95%) sulfuric acid, surface-sterilized for 5 min with 3% of calcium hypochlorite and rinsed a dozen times with sterile deionized water. They were germinated overnight in the dark on agar water and then transferred on Murashige and Skoog [22] macro and micro-elements medium supplemented with Nitsch and Nitsch vitamins (Duchefa Biochemie, The Netherlands), pH 5.6, and 8 g /L bacto-agar (Difco) (MS medium).

### *In vitro* assay for *A*. *evenia* regeneration capability

To establish a callogenesis and regeneration protocol, three types of explants were tested: cotyledons, epicotyls and hypocotyls that were excised from one-week-old *A*. *evenia* plantlets grown under axenic conditions. Explants were cultivated in a solid MS medium containing bacto-agar (8 g /L) supplemented with 30 g /L sucrose, Nitsch and Nitsch vitamins, auxin (NAA = Naphthalene Acetic Acid) and cytokinin (BAP = 6-BenzylAminoPurine) as growth regulators. To define the optimal tissue culture conditions, 3 combinations of NAA and BAP were tested: medium A (0.5 μM NAA, 4.4 μM BAP); medium B (0.05 μM NAA, 4.4 μM BAP); medium C (0.5 μM NAA, 2.2 μM BAP). Media were autoclaved for 20 min at 121°C.

Explants were cultivated at all time in a growth chamber with a temperature of 26°C, 70% hygrometry and with a 16 h photoperiod (50 μE m2^−1^ s^–1^) provided by cool fluorescent tubes (daylight Sylvania 36W/GR0).

Regenerated shoots were rooted by a 1-day-treatment in the MS medium containing 40 g /L sucrose, Nitsch and Nitsch vitamins and 10 μM indole-3-butyric acid (IBA). They were then transferred in the same medium without IBA. Shootlets were subcultured at intervals of 3 weeks.

### Tolerance of A. evenia to hygromycin

Before transformation experiments, the hygromycin sensitivity of *A evenia* was assessed by culturing 50 non-transformed explants in MS medium containing NAA, BA and increasing concentrations of hygromycin: 0, 10, 20, 30 mg/L. Callus growth and bud development were scored after 4–8 weeks.

#### *Agrobacterium tumefaciens* strain culture

*A*. *tumefaciens* bacteria were grown overnight at 28°C in Ag medium [23] containing 50 mg/L kanamycin (Duchefa Biochemie, The Netherlands) and 10 mg/L rifampicin (Duchefa Biochemie, The Netherlands). The bacterial suspension was diluted to approximately 10^8^ cells/ml in MS medium (absorbance = 0.1 at 600nm) before use for genetic transformation of *A*. *evenia*.

#### Transformation procedure

140 explants (110 epicotyls and 30 hypocotyls) were immersed for 1 h in the diluted *A*. *tumefaciens* suspension. After blot-drying between sterile filter papers, explants were placed on solidified MS medium for 3 days. The *A*. *evenia* explants were then rinsed three times, for 1 h each time, with sterile MS medium containing 250 mg/L cefotaxime (Duchefa Biochemie) to eliminate *A*. *tumefaciens*, blotted dry on sterile filter paper and placed on the MS regeneration medium containing 0.5 μM NAA and 4.4 μM BAP, 10 mg/L hygromycin and 250 mg/L cefotaxime. After the first 2 weeks of cultivation, the concentration of hygromycin was increased to 20 mg/L. Two separate controls were included in the experiment. They consisted of 40 untransformed explants (20 epicotyls and 20 hypocotyls) which were placed on MS regeneration medium either with antibiotics (control for antibiotic selection) or without antibiotic (control for regeneration). Explants were transplanted in a freshly prepared medium every 2 weeks and the apparition of contaminations was regularly checked.

#### Selection, regeneration and culture of A. *evenia* transformants

Calli emerging from the cut ends of the explants were selected based on mCherry fluorescence using a macroscope (Nikon AZ100 equipped with Nikon DS-Ri1 camera). They were excised about 8 weeks after cocultivation with *Agrobacterium*, and transferred to glass tubes (2.5 cm in diameter, 20.5 cm in length) containing MS regeneration medium supplemented with antibiotics. Calli were transplanted every 2–3 weeks until some shootlets started to regenerate. Emerging shoots were regularly removed from the calli and transferred to a rooting medium supplemented with hygromycin and cefotaxime. Regenerated plants were transplanted into pots containing BNM (Buffered Nodulation Medium) liquid medium [24] and acclimatised under a mini glasshouse for 1 week before gradual exposure to the growth chamber environment (26°C, 16h photoperiod, 70% hygrometry). Acclimatised plants were transferred to pots containing a mix of potting soil and vermiculite (9:1; v:v). Transgenic plants were then cultivated until the development of seeds that were further harvested.

#### Molecular analysis-based confirmation of the presence of the transgene in regenerated plants

The transgenic nature of plants screened based on their red fluorescence (mCherry) was further confirmed by PCR and DNA sequence analyses. Total DNA was extracted from leaves using the CTAB method [25]. Control DNA from non-transformed plants was included in the experiments to ensure that reagents were not contaminated. The presence of genes encoding hygromycin and mCherry was checked by PCR using specific primers (Hyg-Forward: AAAAGTTCGACAGCGTCTCCGACC& Hyg-Reverse: ATTGTTGGAGCCGAAATCCGCGTGC, mCherry-Forward: GCAAGGGCGAGGAGGATAAC& mCherry-Reverse: CCGTCCTCGAAGTTCATCACGC). To check the absence of *A*. *tumefaciens* contamination, a PCR was also done with primers targeting the gene encoding the bacterial selectable marker kanamycin (Kanbact-Forward: GGGACCACCTATGATGTGG& Kanbact-Reverse: AATCCACATCGGCCAGATCG). Amplified fragments were then sequenced.

## Results and discussion

### Callus induction and regeneration ability of *A*. *evenia*

To develop a plant transformation procedure for a given species, the first step is to test the regeneration ability from different explants and to define the appropriate tissue culture medium. As starting explants, we have selected cotyledons, epicotyls and hypocotyls excised from 1 week old plantlets. We tested 3 regeneration media based on previous reports related to the callogenesis and regeneration from different explants of various species including *Aeschynomene* [18, 26]. All media contained MS-agar supplemented with N&N vitamins and sucrose but differ in their hormonal composition.

**Fig 1:**
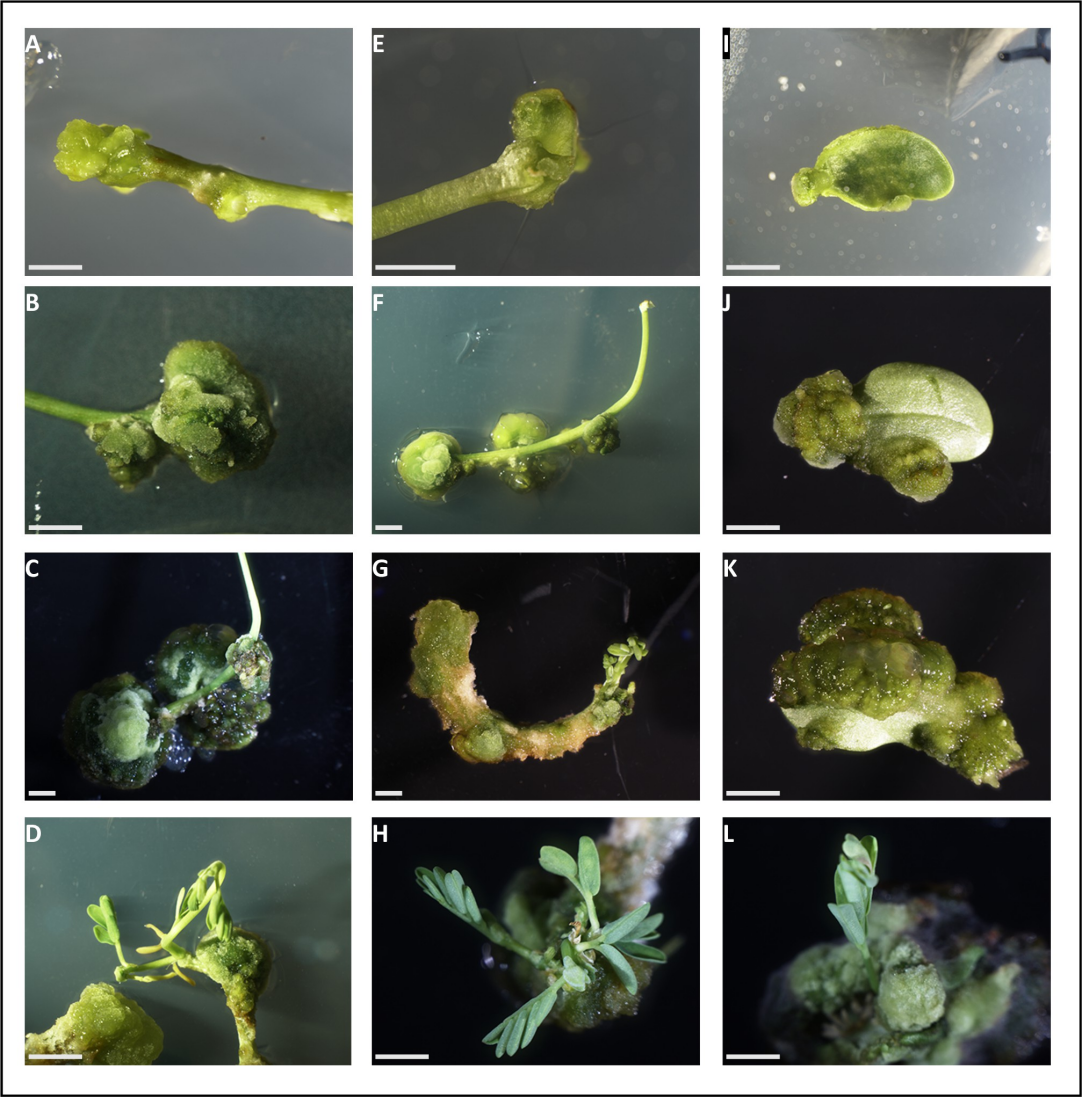
Regeneration of *A*. *evenia* from calli grown from different explants. A-D: Epicotyls; E-H. Hypocotyls; I-L. Cotyledons. Explants were grown in MS regeneration medium for 10 days (A, E, I), 2 weeks (B, F, J), 3 weeks (C, G, K) or 4 weeks (D, H, L). Bars = 2 mm.

After one week of cultivation, we observed the formation of calli from cotyledons, epicotyls, and hypocotyls using various combinations of growth hormones (**Table 1 and Fig 1**). Callogenesis rates were very high especially for media A and C compared to medium B (up to 100%) (**Table 1**). Statistical analyses using the GLM (Generalized Linear Model) followed by a binomial test indicate no significant effect of explant type on callus induction. Regarding the effect of culture media, the rate of callus induction is significantly higher in media A and C compared to medium B (See **S1 File**). The difference observed is likely due to a 10-fold higher concentration of NAA in media A and C compared to medium B pointing to an important role of auxin in the callogenesis of *A*. *evenia*.

**Table 1:** Effect of explant type and media culture composition on callus induction rate in *A*. *evenia*. Cotyledons, epicotyls and hypocotyls explants were tested in MS media containing different hormonal combinations: Medium A (0,5 μM NAA, 4,4μM BAP); Medium B (0,05 μM NAA, 4,4μM BAP); Medium C (0,5 μM NAA, 2,2 μM BAP). Data were compared using the GLM (Generalized Linear Model) followed by a binomial test. For the media culture composition, the rate of callus induction is significantly higher in media A and C compared to medium B. For the explant type, no significant effect was found (See **S1 File**).

	Number of explants with calli /Number of explants*			
	Cotyledons	Epicotyls	Hypocotyls	Total explants
Medium A	39/40 (98%)	15/16 (94%)	15/15 (100%)	69/71
Medium B	29/36 (81%)	14/17 (82%)	12/18 (67%)	55/71
Medium C	49/50 (98%)	25/26 (96%)	25/25 (100%)	99/101

* Contaminated explants were eliminated

After 4 weeks, buds were differentiated on all the induced calli regardless of the type of the explant. Subsequently, shoots continuously proliferated and elongated from the budding calli. A dozen of randomly selected shoots were rooted by a 1-day treatment in the presence of indole-3-butyric acid (IBA), followed by a culture on nutrient medium without growth regulators. All shoots successfully developed roots and plants were generated.

Based on these results, we conclude that *A*. *evenia* has exceptional regeneration capabilities and presumably, other organs of the plant could be used as starting material.

Finally, we encountered no significant challenges in determining the optimal regeneration conditions. Therefore, we decided to proceed with medium C and utilize epicotyls and hypocotyls as the starting material for subsequent experiments. To validate our chosen conditions, we conducted two additional experiments with a smaller sample size, which yielded consistent results.

### Hygromycin resistance allows the selection of transgenic calli

**Fig 2:**
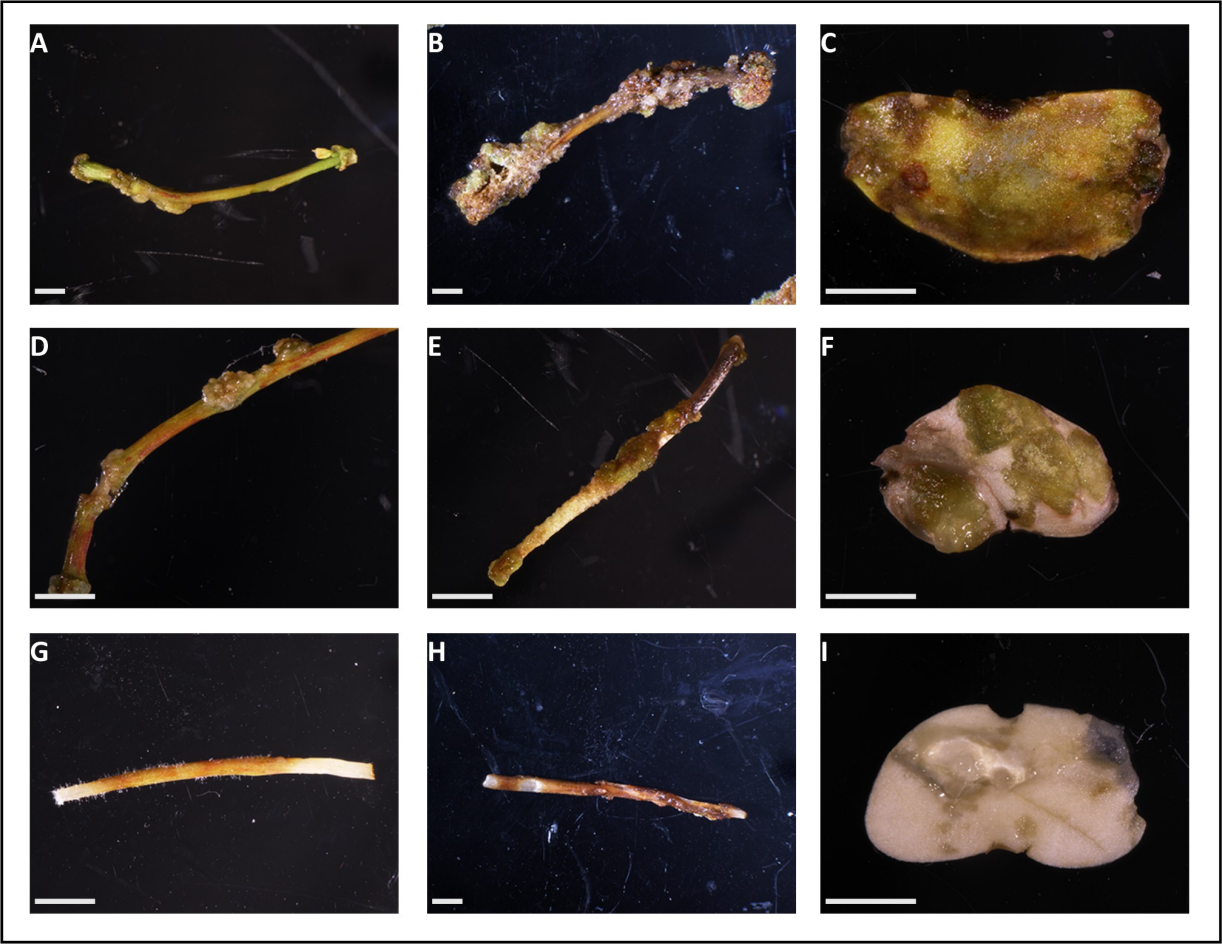
Sensitivity of *A*. *evenia* explants to hygromycin. Epicotyls (A, D, G), hypocotyls (B, E, H) and cotyledons (C, F, I) were grown for 4 weeks in MS regeneration medium containing 10 mg/L (A-C); 20 mg/L (D-F) and 30 mg/L (G-I) of hygromycin. Bars = 2 mm.

The sensitivity of explants to antibiotics is an essential criterion for the selection of transformed cells from which calli can be induced. The most common selectable markers for the transformation of legumes are HPT (hygromycin resistance) and NPTII (kanamycin resistance) gene [27, 28]. According to previous reports, HPT seemed to be a more suitable marker for legumes as it is more stringent and the escape of non-transformed cells is reduced [28–32]. Moreover, kanamycin was previously tested on *A*. *evenia* hairy roots without improving the selection of transgenic roots (F. Cartieaux, personal communication). For these reasons, we chose to test the potential of HPT as a selectable marker for the transformation of *A*. *evenia*. We added increasing concentrations of hygromycin (0 mg/L, 10 mg/L, 20 mg/L and 30 mg/L) to the regeneration medium as described for other legume species [27, 32, 33]. After a four-week cultivation period, we noted significant browning of explants (cotyledons, epicotyls, and hypocotyls) when exposed to 10 mg/L of HPT, which adversely affected callus development (**Fig 2**: photos 2A-2C). This concentration proved to be sufficiently disruptive to the callogenesis process in *A*. *evenia* explants. Furthermore, at 20 and 30 mg/L of HPT, we observed a strong or complete inhibition of callus formation (**Fig 2**: photos 2D-2I). As a result, we determined that 20 mg/L is the appropriate concentration for selecting transformed cells while effectively preventing the proliferation of false positives.

### Transformation and regeneration of *A*. *evenia*

#### Plant transformation

Once the regeneration conditions and the tolerance to selection marker established, we proceeded to the transformation experiment using the *A*. *tumefaciens* strain EHA105. EHA105 is a succinamopine type that has been successfully used for DNA transfer in several legume species including *M*. *truncatula* and Soybean [28, 34, 35].

**Fig 3:**
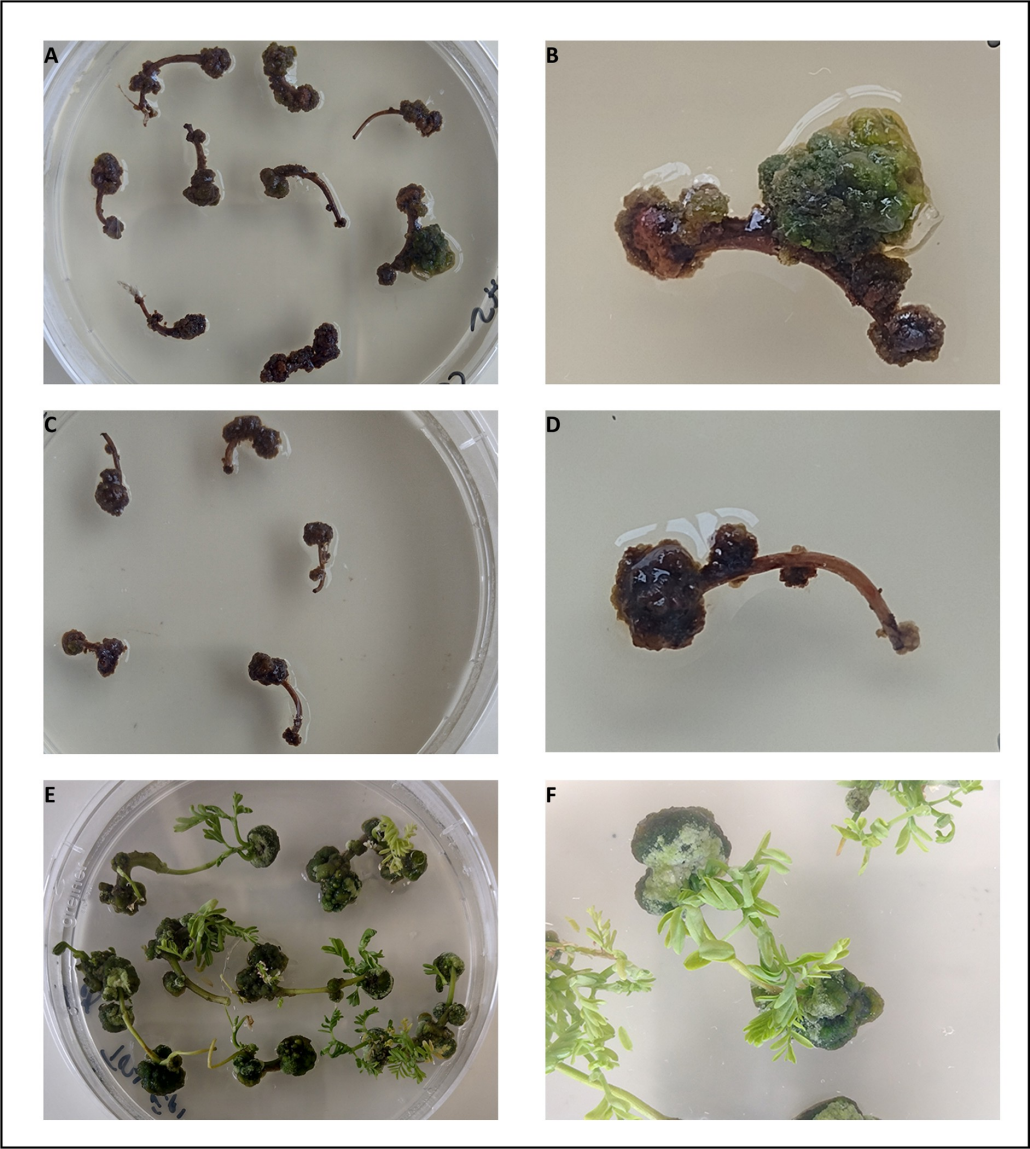
Transformation of *A*. *evenia* with *A*. *tumefaciens* EHA105-pCambia5300-mCherry. A-B Coculture with *A*. *tumefaciens*, C-D Control for antibiotic selection (explants placed in regeneration medium with antibiotics), E-F Control for regeneration (explants placed in regeneration medium without antibiotics). Regeneration control explants 4 weeks after transformation; coculture and control for antibiotic selection 7 weeks after transformation.

As mentioned earlier, because the different explants have similar regeneration potential, we have chosen to use epicotyls as the starting material for the transformation procedure. Additionally, as a precautionary measure, we utilized a smaller number of hypocotyls. We transformed 140 explants (110 epicotyls and 30 hypocotyls). We considered this number sufficient for the transformation experiment as *A*. *evenia* has remarkable regeneration properties. Two weeks after cocultivation, several explants growing in the presence of hygromycin initiated the formation of green calli. Non-transformed explants cultivated on medium without antibiotics (control for regeneration) rapidly developed green calli. Calli continued to grow on plates for 6 weeks. Those obtained from cocultivated explants grew at a slower pace compared to the regeneration controls. They also showed variations in size and growth rate, likely resulting from different transformation events. **(Fig 3)**. Control explants cultivated with hygromycin in the absence of *A*. *tumefaciens* (control for antibiotic selection) produced smaller and brown calli **(Fig 3)**.

**Fig 4:**
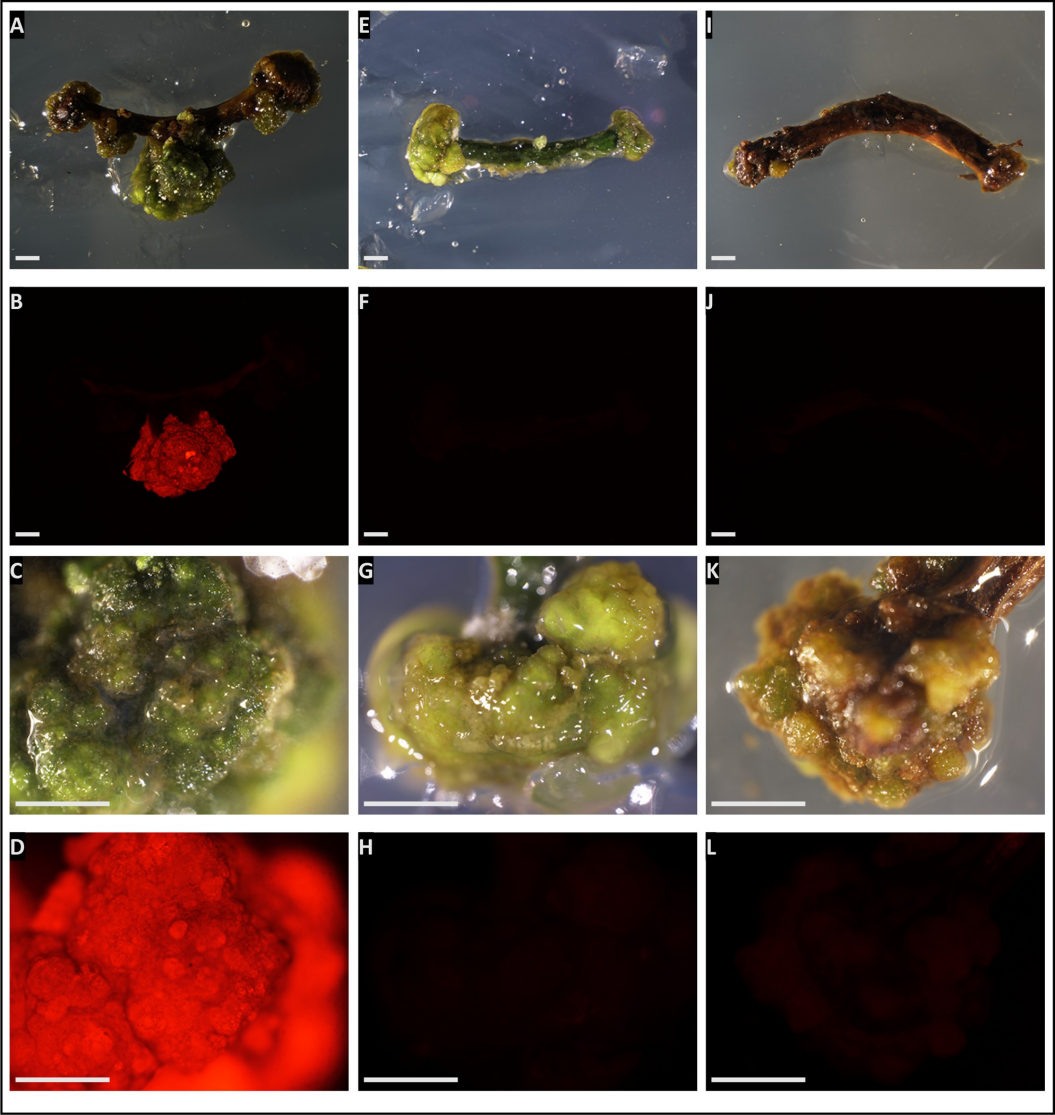
mCherry fluorescence observed in *A evenia* calli under different conditions. Panels A-D display transformed calli obtained through cocultivation with *A*. *tumefaciens* carrying the Pro35S:mCherry construct. Panels E-H show calli obtained without *A*. *tumefaciens* and without hygromycin (control for regeneration), while panels I-L represent calli obtained with hygromycin (control for antibiotic selection). Images A, C, E, G, I, and K were captured under white light, while images B, D, F, H, J, and L exhibit the red fluorescence under green light and with adequate red filter. The images displayed in panels B, F, and J were captured using identical settings (in particular the exposure time), as well the images in panels D, H, and L. Bars = 2 mm.

After 6 weeks of cocultivation, a total of 77 (55%) calli (47 from epicotyls and 30 from hypocotyls) were formed (**Table 2**). Fifty-three (69%) out of them (40 from epicotyls and 13 from hypocotyls) showed high mCherry fluorescence (**Table 2 and Fig 4**). Thus, the transformation frequency was 85% for epicotyls and 43% for hypocotyls (**Table 2**). Similar results were obtained in a second experiment carried out with 140 epicotyls and hypocotyls (70 each). Based on these results we conclude that our protocol enables the generation of transgenic *A*.*evenia* calli with a remarkable efficiency.

**Table 2:** Genetic transformation of *A*. *evenia* using epicotyls and hypocotyls as starting explants. Fluorescent calli were selected, and each callus continuously produced shoots (1–12 shoots) that were subsequently rooted. Independent lines were obtained from these rooted shoots.

	Epicotyls	Hypocotyls	Total
Number of explants	110	30	140
Number of calli	47 (42%)	30 (100%)	77 (55%)
Number of fluorescent calli	40 (85%)	13 (43%)	53 (69%)
Number of fluorescent calli with shoots	23 (58%)*	4 (31%)*	27 (51%)*
Number of rooted shoots issued from different fluorescent calli	63	14	79
Total number of plants obtained from rooted shoots	58	11	69
Number of independent lines	15	3	18

* The percentage is calculated on the number of fluorescent calli

### Regeneration of transgenic plants

**Fig 5:**
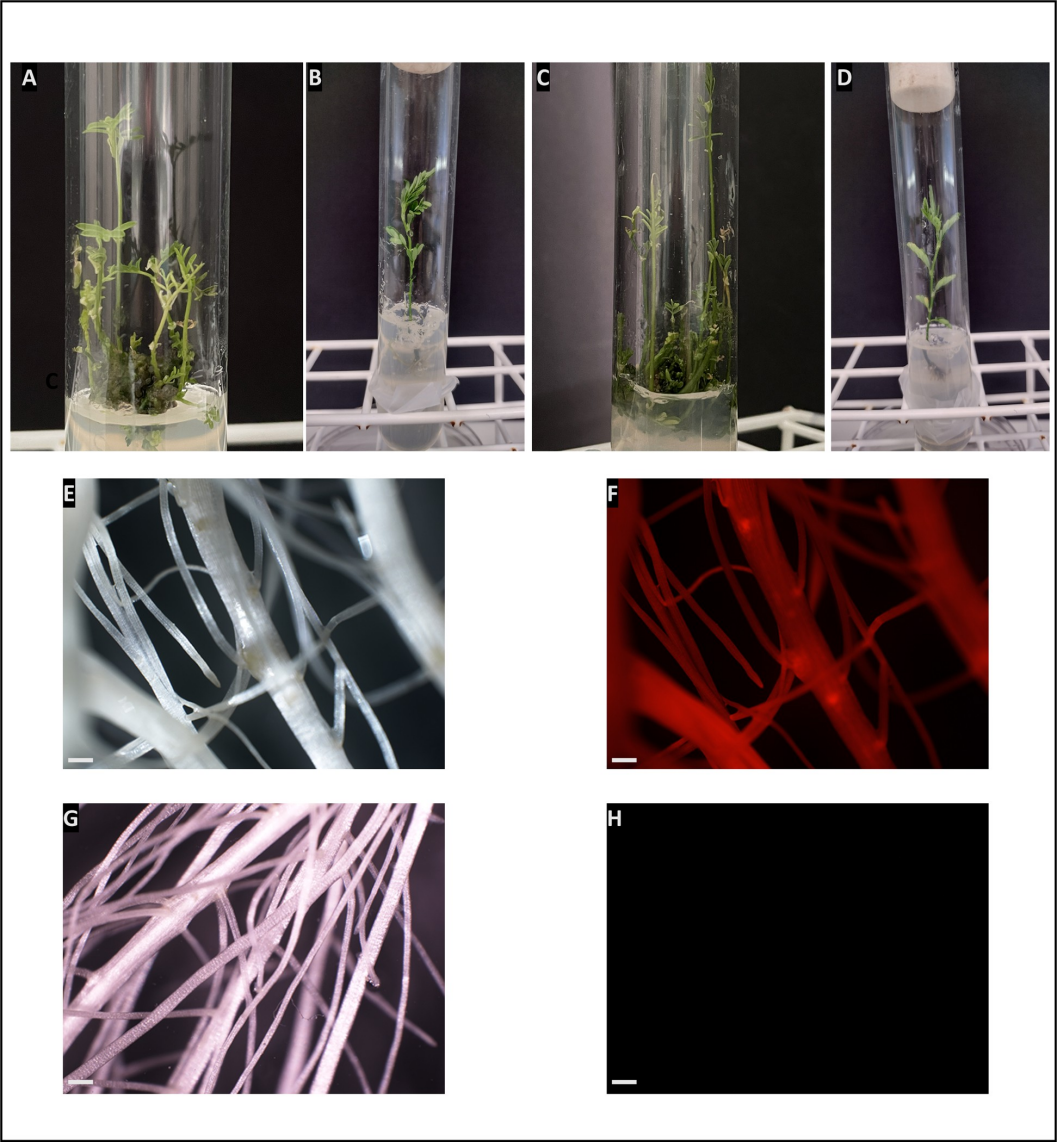
Regeneration of transgenic *A*. *evenia*. A and C, Shoot proliferation from transgenic calli and control non-transgenic calli (respectively). B and D, Rooting of transgenic shoots and control non-transgenic shoots (respectively). E and F. Transgenic roots observed under white light (F) and under green light and with adequate red filter (E). G and H. Control non-transgenic roots under white light (G) and under green light and with adequate red filter (H). Image E shows red fluorescence. Images displayed in panels F and H were captured using identical settings. Bars = 2 mm.

After eight weeks of cocultivation, the highly fluorescent calli, along with six non-transgenic calli from the regeneration control set, were transferred to tubes for shoot induction. The remaining calli were discarded (**Table 2**). The growth of calli was uneven. Larger ones were divided into two pieces during transplantation into new tubes. Shoot induction began ten weeks after cocultivation, and the majority of budding calli exhibited continuous stem proliferation (**Fig 5**). Among the fluorescent calli, 23 (58%) derived from epicotyls and 4 (31%) from hypocotyls displayed the ability to develop shoots (see **Table 2**). Certain calli consistently produced a significant number of shoots (6–12), while others formed fewer shoots (1–5).

Shootlets were excised from the transgenic calli and the non-transgenic control calli when they reached 2–3 cm long and rooted after a treatment with indole-3-butyric acid (IBA). Roots appeared 1 week later in most shoots (nearly 90%) while a small number remained unrooted and turned brown **(Table 2)**. Finally, we were able to regenerate 58 plants from 18 independent calli that were hygromycin resistant and showed mCherry fluorescence (15 from epicotyls and 3 from hypocotyls). Six plants were also regenerated from non-transgenic control calli.

Regenerated plants were transferred in a hydroponics system and placed in a growth chamber (**S1 Fig**). From there, 28 individuals were further transferred to a glasshouse, where they were placed in a solid substrate and exposed to a 12-hour photoperiod to induce flowering. After four weeks, all the lines flowered (**S1 Fig**) and subsequently produced seeds. Notably, in terms of flowering kinetics and seed production, no discernible differences were observed between the transformed and the non-transformed control plants.

In addition, we tested the ability of transgenic plants to develop root nodules. A group of twelve individuals were inoculated with the photosynthetic *Bradyrhizobium* sp strain ORS278 [4]. All plants were able to nodulate similarly to non-transgenic *A*. *evenia*.

#### Molecular analysis of regenerated plants

To confirm the presence of the transgene in the genome of the transgenic rooted plants, PCR analyses were done on 16 transgenic individuals regenerated from 16 independent calli. Two non-transgenic plants were also included as negative controls. Genomic DNA extraction followed by the amplification with hygromycin and mCherry specific primers gave fragments at expected size for all plants tested (**S2 Fig**). A PCR was also performed with primers specific to bacterial kanamycin gene that is present in the binary vector but not in the T-DNA and is not transferred to the plant. For all transgenic plants, no fragment was amplified (**S2 Fig)** suggesting that the amplifications obtained with mCherry and hygromycin specific primers were not the result of *A*. *tumefaciens* contamination. Finally, amplified DNA fragments were sequenced, and as expected, all of them correspond to the sequences of either the hygromycin or mCherry gene fragments.

### A genetic transformation method for *A*. *evenia*

**Fig 6:**
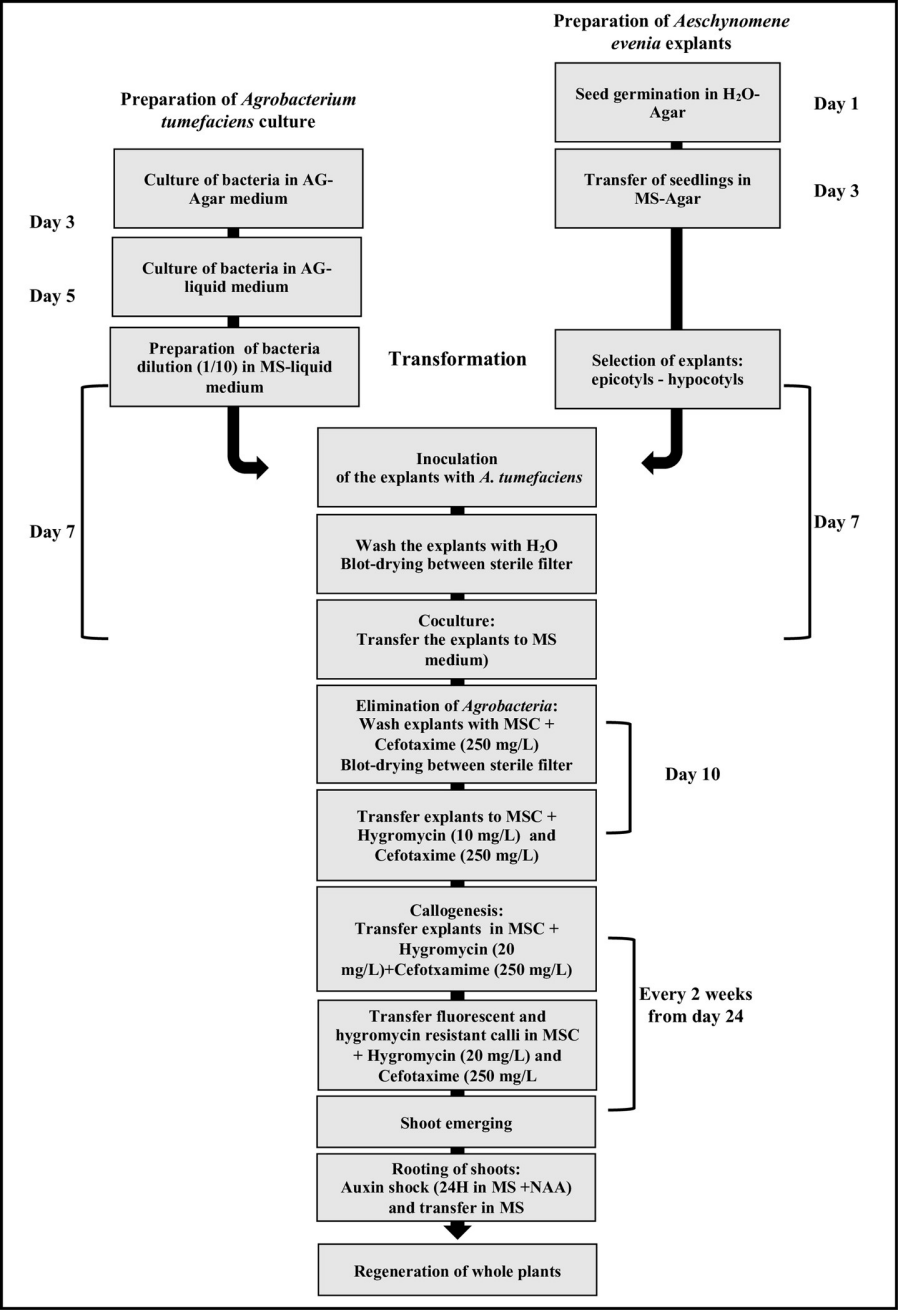
Summary of the *A*. *tumefaciens* transformation procedure for *A*. *evenia*. Whole transgenic plants can be regenerated with a 4-month timeline. Culture media used: AG; MS (MS+NN vitamins+sucrose), MSC (MS+NN+sucrose+NAA+BAP).

In this study we report the development of a highly efficient *in vitro A*. *tumefaciens*-mediated transformation and regeneration system for *A*. *evenia* (**Fig 6**). We have shown that one growth medium containing a combination of NAA and BAP was sufficient to induce callogenesis from various explants and subsequently the production of shoots in only 4 weeks. On the other hand, we have found that *A*. *tumefaciens* strain EHA105 was sufficiently virulent to yield a high number of transformed calli while the use of hygromycin as a selectable marker was enough efficient to prevent the induction of budding calli from non-transformed material. Data obtained highlight the tremendous regeneration ability of *A*. *evenia*.

The stable transformation method developed for *A*. *evenia* allows the production of high number of independent transformants within only 4 months. This protocol could be improved by optimizing some parameters defined elsewhere: medium composition, virulence of the bacterial strain, cocultivation time etc.… [28, 36]. It constitutes a good basis and can be adapted to other *Aeschynomene* species.

Compared to the hairy-root procedure based on *A*. *rhizogenes* which was already used for *Aeschynomene* gene function analyses [15, 16], the transformation *via A*. *tumefaciens* presents several advantages. The main of them being the ability to conserve the seeds of the transgenic lines, which may facilitate long-term genetic studies. Another added value of the stable transformation is that each transgenic line is issued from one transformation event and exhibits a homogeneous phenotype. Consequently, the number of independent lines necessary for functional analyses is much lesser compared to hairy-root method. conversely, in the case of *A*. *rhizogenes* mediated transformation, each composite plant contains a mix of transgenic roots resulting from independent co-transformation events often leading to a heterogeneous phenotype. As a result, a larger number of transgenic plants is needed. Thus, thanks to the stable transformation, gene function studies, particularly those based on genome editing approaches (CRISPR) are greatly facilitated.

For *Aeschynomene*, application of this new process should help to decipher the molecular mechanisms governing the NodF-independent symbiosis. Indeed, it is now conceivable to introduce reporter genes in a mutant genetic background (for example promoter:GUS gene fusions or molecular sensors such as the calcium sensor GECO [37]. It is also possible to study genes controlling nodulation events from shoots such as SUNN, a gene involved in the autoregulation of nodulation [38]. Furthermore, powerful approaches like loss-of-function, gain-of-function or complementation can be carried out without the bias provoked by *A*. *rhizogenes* strains which harbor the ROL virulence genes whose expression is necessary for the induction of hairy-root but alters the phenotype of transgenic roots [17]. This is particularly true for genes involved in hormone signalling like the cytokinin pathway. Finally, since *Aeschynomene* has the property to form stem nodules, this will enable the investigation of such original nodulation process which cannot be achieved using the hairy-root method.

## Conclusion

The development of an efficient *A*. *tumefaciens*-mediated transformation method for *A*. *evenia* offers a new range of possibilities to perform functional studies This technical advance will greatly facilitate the deepen characterization of key genes involved in the NodF-independent symbiotic signaling pathway. Moreover, this protocol could be adapted for *Aeschynomene patula*, a closely related species that is exclusively nodulated through NodF-dependent pathway. Both *A*. *evenia* and *A*. *patula* use a similar intercellular infection process but differ in their requirement for Nod factors to trigger nodulation. Thus, the availability of such transformation procedure will offer a unique opportunity to compare the NodF-independent *versus* the NodF-dependent processes by carrying out reciprocal ectopic expression and gain-of-function experiments of host symbiotic genes specific to each process. Finally, this will provide important clues on the specificity and evolution of genes mediating the NodF-independent symbiosis.

## Supporting information

S1 File Statistical analyses. (DOC)

S1 Fig Culture of transgenic *A*. *evenia*. A. Transgenic plants grown in hydroponics system. B, C. Transgenic plants flowering in the greenhouse.
(TIF)

S2 Fig Confirmation of genetic transformation of *A*. *evenia* by molecular analysis. A. PCR using mCherry specific primers. B. PCR with hygromycin specific primers. C. PCR with kanamycin specific primers (present in pCambia5300 mCherry plasmid but not in the T-DNA). 1. Individuals of *A*. *evenia* transgenic lines. 2. Non-transgenic *A*. *evenia* control plants. 3. Negative PCR control without DNA. 4. Positive PCR control 1 (pCambia5300 plasmid containing the Pro35S:mCherry construct). 5. Positive PCR control 2 (*A*. *tumefaciens* EHA105 with the pCambia5300 plasmid containing the Pro35S:mCherry construct). Amplification of mCherry and hygromycin gene fragments is apparent for transgenic plants and positive controls but not for wild type plants and the negative control. The kanamycin gene fragment was amplified only for the pCambia5300 plasmid DNA and bacteria containing this plasmid.
(TIF)
