# Supplementary material for: Setting up Agrobacterium tumefaciens-mediated transformation of the tropical legume Aeschynomene evenia, a powerful tool for studying gene function in Nod Factor-independent symbiosis
Source: PLoS One. 2024 Apr 16;19(4):e0297547. doi: 10.1371/journal.pone.0297547 (PMC11020691; doi:10.1371/journal.pone.0297547)
Supplement: S1 File — (DOC) [file pone.0297547.s001.doc]

**S1 Supporting information**

**Statistical analyses**

Statistical analyses were performed with RStudio software (version 1.4.1717) to investigate the effect of explant type and media culture composition on callus induction rate. Data were subjected to GLM (Generalized Linear Model) followed by a binomial test for the effect of each parameter (medium and explant). GLM is a statistical method commonly used to model binary or count data (Nelder and Wedderburn, 1972).

The statistical test indicates that there is only an effect of the medium on callus induction, and no interaction between the medium and explant type.

|  | Df | Deviance | AIC | LRT | Pr(>Chi) |  |
| --- | --- | --- | --- | --- | --- | --- |
| <none> |  | 3.8533 | 31.354 | 31.354 |  |  |
| Medium | 2 | 28.3290 | 51.830 | 24.4758 | 4.843e-06 | *** |
| Explant | 2 | 4.2994 | 27.800 | 0.4461 | 0.8001 |  |

- For the media culture composition, the rate of callus induction is significantly higher in media A and C compared to medium B.

| Medium A | 100*round(binom.test(c(69,2))$conf[1:2],3)90.2 99.7 |
| --- | --- |
| Medium B | 100*round(binom.test(c(55,16))$conf[1:2],3)  66.0 86.5 |
| Medium C | 100*round(binom.test(c(99,2))$conf[1:2],3) 93.0 99.8 |

- For the explant type, no significant effect was found between cotyledons, epicotyl and hypocotyl (p=0,481).

| Cotyledon | 100*round(binom.test(c(117,9))$conf[1:2],3)*10086.9 96.7 |
| --- | --- |
| Epicotyl | 100*round(binom.test(c(54,5))$conf[1:2],3)*100  81.3 97.2 |
| Hypocotyl | 100*round(binom.test(c(52,6))$conf[1:2],3)*100  78.8 96.1 |

**Reference:**

Nelder, JA, Wedderburn RW. (1972). "Generalized linear models". Journal of the Royal Statistical Society, Series A. Royal Statistical Society. 1972. 135 (3): 370–384. doi:10.2307/2344614.
