# Supplementary figures and images for: Setting up Agrobacterium tumefaciens-mediated transformation of the tropical legume Aeschynomene evenia, a powerful tool for studying gene function in Nod Factor-independent symbiosis
Source: PLoS One. 2024 Apr 16;19(4):e0297547. doi: 10.1371/journal.pone.0297547 (PMC11020691; doi:10.1371/journal.pone.0297547)

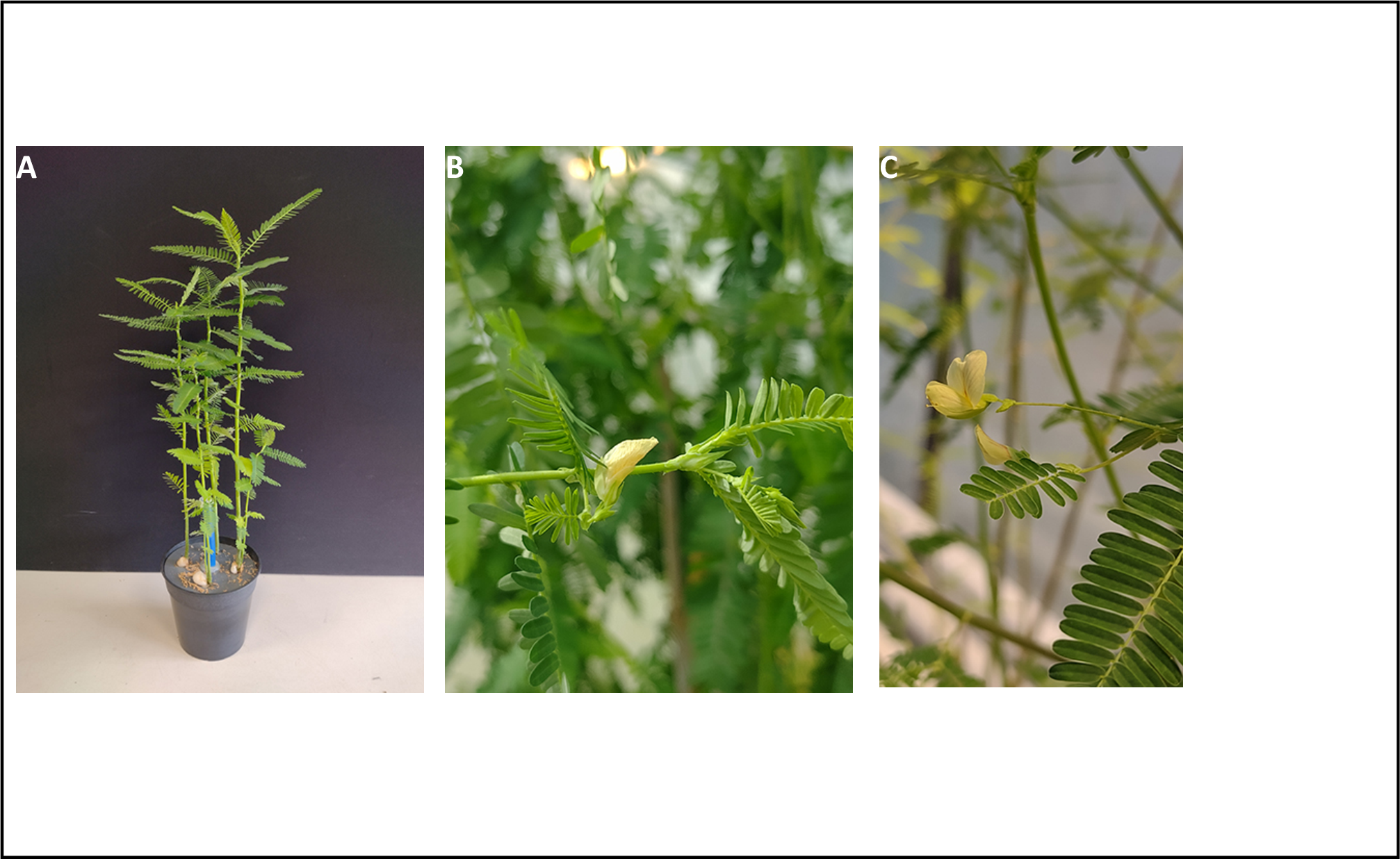

Supplement: S1 Fig — A. Transgenic plants grown in hydroponics system. B, C. Transgenic plants flowering in the greenhouse. (TIF) [file pone.0297547.s002.tif]

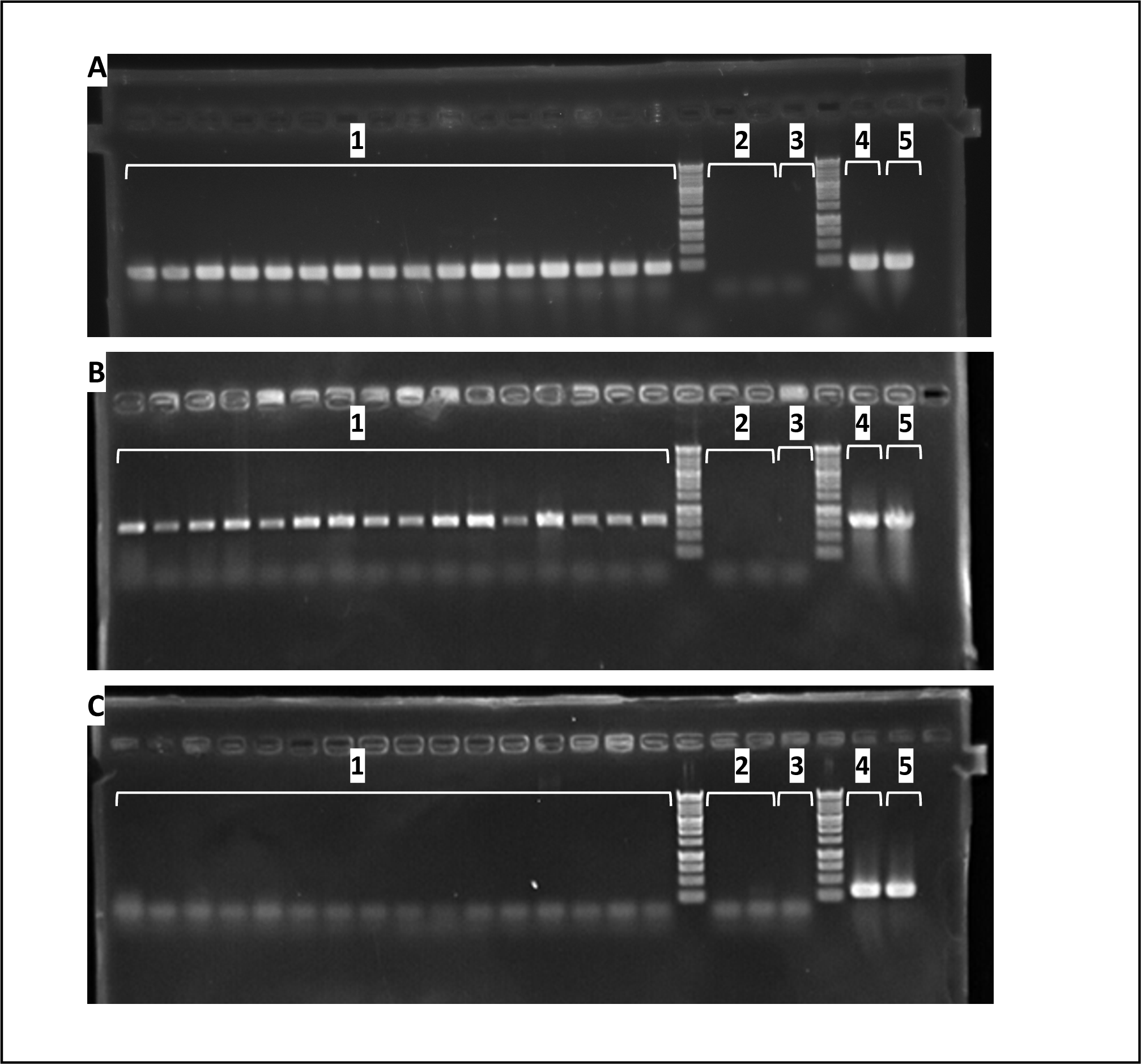

Supplement: S2 Fig — A. PCR using mCherry specific primers. B. PCR with hygromycin specific primers. C. PCR with kanamycin specific primers (present in pCambia5300 mCherry plasmid but not in the T-DNA). 1. Individuals of A. evenia transgenic lines. 2. Non-transgenic A. evenia control plants. 3. Negative PCR control without DNA. 4. Positive PCR control 1 (pCambia5300 plasmid containing the Pro35S:mCherry construct). 5. Positive PCR control 2 (A. tumefaciens EHA105 with the pCambia5300 plasmid containing the Pro35S:mCherry construct). Amplification of mCherry and hygromycin gene fragments is apparent for transgenic plants and positive controls but not for wild type plants and the negative control. The kanamycin gene fragment was amplified only for the pCambia5300 plasmid DNA and bacteria containing this plasmid. (TIF) [file pone.0297547.s003.tif]
